# Painful Life Events are Associated with Brain Aging in Those High in Catastrophizing: An Observational Study

**DOI:** 10.1093/geroni/igaf122.3633

**Published:** 2025-12-31

**Authors:** Josue Cardoso, Elizabeth Losin

**Affiliations:** Pennsylvania State University, University Park, Pennsylvania, United States; Pennsylvania State University, University Park, Pennsylvania, United States

## Abstract

Chronic pain becomes more common and burdensome with age, and past work has linked brain aging with chronic pain. Nonetheless, it remains unclear whether painful life experiences and pain coping styles are related to brain age in individuals who do not yet have chronic pain. In this secondary analysis, we examined associations between pain coping, painful life experiences, and brain age in a sample of 97 healthy adults between ages 18 and 54. Brain age (brain-PAD) was estimated using a machine learning model trained on T1-weighted MRI data (brainageR). Given the overlap in variables collected for the parent study, Stochastic Search Variable Selection was used to identify important predictors of brain age, as this exploratory approach handles correlated data and model uncertainty more robustly than traditional methods. A moderated mediation model, informed by past literature, was then specified to assess relationships among variables. We found that catastrophizing significantly moderated the link between the number of major painful life events and brain aging. Specifically, as the number of major painful life events increased, individuals who tended to catastrophize more also tended to have higher fear of pain, which, in turn, was related to higher brain age. These findings suggest that maladaptive coping may impact brain aging even in individuals without chronic pain. Pain catastrophizing, particularly, is highlighted as a potential target for intervention. Lastly, the present findings may inform future mechanistic and multilevel longitudinal research aimed at further disentangling the complex relationships between brain aging and acute, subacute and chronic pain.

